# Electrosurgical Smoke: Ultrafine Particle Measurements and Work Environment Quality in Different Operating Theatres

**DOI:** 10.3390/ijerph14020137

**Published:** 2017-01-30

**Authors:** Francesco Romano, Jan Gustén, Stefano De Antonellis, Cesare M. Joppolo

**Affiliations:** 1 Politecnico di Milano, Dipartimento di Energia, via Lambruschini, 4, 20156 Milan, Italy; stefano.deantonellis@polimi.it (S.D.A.); cesare.joppolo@polimi.it (C.M.J.); 2 Civil and Environmental Engineering/Building Services Engineering, Chalmers University of Technology, SE-41296 Gothenburg, Sweden; jan.gusten@chalmers.se

**Keywords:** operating theatre, electrosurgical tool, surgical smoke, ultrafine particles, work environment quality, ventilation principles

## Abstract

Air cleanliness in operating theatres (OTs) is an important factor for preserving the health of both the patient and the medical staff. Particle contamination in OTs depends mainly on the surgery process, ventilation principle, personnel clothing systems and working routines. In many open surgical operations, electrosurgical tools (ESTs) are used for tissue cauterization. ESTs generate a significant airborne contamination, as surgical smoke. Surgical smoke is a work environment quality problem. Ordinary surgical masks and OT ventilation systems are inadequate to control this problem. This research work is based on numerous monitoring campaigns of ultrafine particle concentrations in OTs, equipped with upward displacement ventilation or with a downward unidirectional airflow system. Measurements performed during ten real surgeries highlight that the use of ESTs generates a quite sharp and relevant increase of particle concentration in the surgical area as well within the entire OT area. The measured contamination level in the OTs are linked to surgical operation, ventilation principle, and ESTs used. A better knowledge of airborne contamination is crucial for limiting the personnel’s exposure to surgical smoke. Research results highlight that downward unidirectional OTs can give better conditions for adequate ventilation and contaminant removal performances than OTs equipped with upward displacement ventilation systems.

## 1. Introduction

### 1.1. Electrosurgical Devices

Operating theatres (OTs) are sensitive environments for indoor air quality (IAQ) in healthcare premises. The extensive use of electrosurgical tools (ESTs) during surgical procedures generates a large quantity of surgical smoke. Surgical smoke is acknowledged as a work environment and occupational health problem, as a result of its unpleasant odor and the possibility of obstructing the view of the surgical site. Besides these comfort and productivity issues, surgical smoke can cause health problems [1] to the surgical staff. Surgical smoke is generated by all ESTs, such as monopolar, bipolar and argon diathermy and other devices which use high-frequency alternating current for tissue dissection or cauterization [2]. ESTs are routinely used tools during surgical operations. Monopolar EST refers to an arrangement of a single small electrode placed at the tip of a surgical instrument that delivers focused alternating current to a target tissue for the desired surgical effect [2,3,4], generally cutting or cauterization. A second large return electrode is placed in a remote side of the patient body and is connected to the current generator acting also as the electrical ground. In this case, the thermal effect remains localized to the probe’s tip as the current rapidly enters the body [2]. In the monopolar ESTs, the electrical current flows from the active electrode to the human body, then back to the electrical generator through the second electrode. In bipolar arrangement, the EST forceps has two tine electrodes; the high-frequency electrical current flows through the two tiny electrodes with the tissue in between. argon diathermy ESTs use a flow of inert argon gas which is ionized with high-frequency alternating current ducted by the active electrodes. Here, a plasma cloud is formed and sparks or electric arcs operate over the target tissue without contact. Argon diathermy ESTs could have some advantages, e.g., reducing blood loss, causing less tissue damage, producing less smoke and odor, improving healing and lowering infection risk [5].

All ESTs heat the target cells up to the boiling point causing the cell membranes to rupture [4,6,7] and generate surgical smoke containing fine and ultrafine particles and gases. The plume generated is visible and odorous. Surgical smoke is composed of approximately 95% water (liquid phase or steam) and 5% organic vapors and cellular debris in form of particulate matter (PM) [8]. The energy released by ESTs produces many complex compounds that may be harmful to health including polycyclic aromatic hydrocarbons (PACs). The particulate matter consists of chemicals, blood, tissue particles and bacteria. It has been demonstrated that electrosurgical devices may produce high quantities of ultrafine particles (UFP) and fine particles (FP) with diameters mostly in the range from 0.01 μm up to 1 μm [9,10,11]. Larger particle diameters are also produced, and particle peak concentrations are just close to the target tissue [12]. Previous studies have indicated that ESTs produce particles with a mean aerodynamic diameter of 0.07 μm, laser-produced particles have a mean diameter of 0.31 μm while ultrasonic scalpels produced particles range from 0.35 to 0.65 μm [13,14].

The surgical smoke generated by ESTs is released into the OT environment with quite high velocities and the airborne contaminants can be spread, by convection and diffusion, quite far from the target tissue in a relatively short time [15]. Studies conducted by Brandon & Young [16] are showing how surgical smoke can spread everywhere in an OT suite with time-peak concentrations when ESTs are in use but with concentration levels remaining high long after their usage. Few previous studies have focused on the monitoring of the particle concentration generated by ESTs [17], while other works focused on the characterization of the volatile organic compounds in diathermy plumes [18,19].

### 1.2. Surgical Smoke and Workplace Health

Airborne particles with a diameter less than 10 μm are inhalable [8] and may deposit in the respiratory tract, while particles with a diameter less than 2.5 μm precipitate in the alveolar region of the lungs and this could induce more adverse effects [20,21]. UFPs, which have a diameter of less than 0.1 μm, can more deeply penetrate in the respiratory system. They have a high deposition rate in the low respiratory tract, as stated by [11,22], and thus a higher potential than larger particles in causing health risks [23].

The potential health risks related to the exposures and inhalation of surgical smoke have been linked to acute adverse health effects in exposed healthcare workers [8,9,24,25,26,27], including: eye, nose and throat irritation, headache, cough, and nasal congestion [3,22,28,29]. Surgical smoke has been shown to induce acute and chronic inflammatory changes (e.g., emphysema, asthma, chronic bronchitis) in the respiratory tract of animal models [30,31]. Scientific data on long-term effects of exposure to surgical smoke are unsystematic and scarce. A study notes that chronic exposure to fine particulate air pollution is associated with an increased incidence of cardiovascular disease and death among postmenopausal women [32].

The potential risk of health disease transmission to medical and perioperative staff in OTs via viral and bacterial pathogens present in surgical smoke has been studied by many groups [30,33,34,35,36]. Detailed measurements carried out by Hansen et al. [37] bring up a dramatic increase in concentration of both FPs and UFPs when using electrosurgical devices, and in particular argon diathermy; these findings were confirmed also by Scaltriti et al. [24], and Lundblad and Nilsson [38]. Hill et al. [39] evaluated the surgical smoke generated by a simulated diathermy operation using porcine tissue, quantifying and confirming the potential exposure risk for OT staff members.

The health and comfort impacts of surgical smoke are a relevant, and still growing, concern for perioperative team members and their workplace safety [1,10,11,13,28,40,41]. Surgeons usually experience a higher surgical smoke exposure level than other surgical team members who are exposed for even longer time periods in OT. This higher exposure level seems to be attributable to the fact that they stand closer to the surgical table in the zone where ESTs generate surgical smoke, e.g., within five feet from source generation [28,42]. Let us highlight that the surgeon and the medical staff breathing zone, as denoted by the American Society of Heating, Refrigerating and Air-Conditioning Engineers (ASHRAE) [43], encompasses most of the surgical smoke contamination generated by ESTs. A recent report by the Occupational Safety and Health Administration (OSHA) estimated that almost 500,000 healthcare workers including surgeons, nurses, anesthesiologists, surgical technologists, and others are exposed to laser or electrosurgical smoke [1].

### 1.3. Personal Protective Equipment (PPE), Local Smoke Evacuators and General Ventilation

Preventive measures, i.e., local exhaust ventilation (LEV) devices, and personal protective equipment, such as protective masks, can be used in OTs to limit inhalation and exposure to surgical smoke.

LEV devices, used as close as possible to the airborne contaminant source, can provide an effective smoke evacuation. Fixed or portable evacuator equipment is often present in OTs, and ESTs often do have even their own integrated smoke evacuation system. The use of smoke evacuators is quite commonly recommended to surgeons, but they are sometimes difficult to incorporate into the surgical procedure and could interfere with surgery activities. The LEV devices could be large and loud, and therefore they are seldom used, leaving healthcare workers exposed to smoke hazard [6,17,18,39]. Let us highlight that to capture the smoke close to the instrument is somewhat conflicting with the tool working over a surface, markedly with argon diathermy ESTs. The effectiveness of smoke evacuators could be negatively affected by high velocities of aerosol jet sprayed by the contaminant source or by interaction with the air velocities due to the OT ventilation system. Finally, the LEV devices should be (but are not always) equipped with Ultra Low Penetration Air (ULPA) filters in order to avoid reintroducing any UFPs into the OT environment, due to the air recirculation system [8].

Standard surgical masks adopted as PPE by surgical teams are ineffective in filtering the UFP and the smallest FP fraction of surgical smoke. High filtration surgical masks, although offering more effective smoke protection, are not user-friendly and may increase personnel discomfort [27,44]. Both standard and high efficiency surgical masks can easily be penetrated by airborne particles and microorganisms less than 0.1 μm in size, thus offering an incomplete health protection to surgeons and personnel [8,11,13].

As stated by Steege [28], professional and governmental organizations, i.e., Occupational Safety and Health Administration (OSHA) [1], National Institute for Occupational Safety and Health (NIOSH) [45], Association of periOperative Registered Nurses (AORN) [46,47], Association of Surgical Technologists (AST) [48], the American Society for Laser Medicine and Surgery Laser Safety Committee (ASLMS) [49], the American National Standards Institute ANSI Z136.3-2005 (introduced in 2005, updated in 2011) (ANSI) [50], recommend the use of LEV for the protection of workers from surgical smoke hazard. Thus, surgical smoke is widely recognized as an issue of concern, and all the available control options, i.e., personal protective equipment (PPE), local smoke evacuators and general ventilation, are used separately or in combination; however, there is no OSHA standard for a specific laser and electrosurgery plume hazard [28].

Therefore, the engineering control of airborne contamination represents the preferred approach to mitigate workplace exposure and hazards [10,51] and a well-designed and adequately performing OT general ventilation system seems the main way of reducing the smoke concentration and the surgical team exposure. General ventilation principles used in OT are the vertical (or sometimes, horizontal) unidirectional airflow, the upward displacement system, and the mixing system. The ventilation principles adopted, and their design and operation parameters, determine the contaminant control results. The performances of OT ventilation systems have been widely studied, in order to highlight their suitability and to assess the effects of the many parameters related to airborne contaminants control, e.g., airflow pattern, airflow rate, number of personnel present, adopted filtration stages, air temperature and humidity, pressurization, and frequency of door opening [9,12,18,20,22,23,52,53,54].

Balocco et al. [55] and Sadrizadeh [56], by means of numerical simulation and experimental tests, studied the influence of personnel presence and of their gowning and they indicated the importance both of positions occupied and of clothing system worn. Smith [54] and Villafruela [57] studied the effects of door opening in unidirectional airflow ventilated OT showing how it affects the indoor contaminant concentrations. The vertical unidirectional airflow systems, characterized by large airflow volumes, always offer better ventilation performance and cleaner air conditions both in the personnel breathing zone and inside the critical zone occupied by instrumentation and medical staff, near to the surgical table [43,58,59].

The problem in creating an effective evacuation for surgical smoke in OTs leads to different prerequisites for ventilation principles and different surgical routines within an OT. A continuous and local monitoring of UFPs during real surgical activities in OTs in the critical area of the surgical table can better describe how different ventilation principles behave in reducing the level of UFP contamination within the critical area of the OT environments where medical staff operates.

### 1.4. Research Aim

In this research work, the surgical smoke concentrations generated during a number of real surgical operations have been experimentally monitored together with data inherent to the number of medical staff members, the type of ESTs used, the type of surgical activity and the ventilation principle in use, e.g., hybrid theatres with unidirectional downward airflow (UDV) and upward displacement airflow (UWD) ventilation systems.

In particular, the research aim is to evaluate which of the two ventilation principles under evaluation may guarantee a better protection from personnel contamination and exposure to the UFPs generated by ESTs in similar surgical routines, offering a good environment quality during working conditions. The measurements carried out focused on the area close to the surgical table where the activities and the exposure of medical staff and surgeons are concentrated during surgeries, as stated also by Steege [28].

## 2. Materials and Methods

### 2.1. Operating Theatres Environment

Measurements have been carried out during normal surgical operations in five OTs: four of them are common OTs and they are equipped with upward displacement airflow (UWD) systems, the fifth is a hybrid-type OT and is using the unidirectional downward airflow (UDV) principle. Hybrid-type OTs are larger than common ones in order to have surgical procedures and, in the same room, also have diagnostic and imaging devices. Owing to the combined functions, the hybrid OTs are complex working environments where surgeons, nurses, anesthesiologists, and technicians work together, carrying out surgical operations, e.g., cardiac, vascular, and neurological ones.

Main technical data of the four OTs, all similar in dimensions and technical characteristics, equipped with UWD ventilation, and of the larger and more complex fifth hybrid OT adopting UDV ventilation are shown in Table 1. Supply air is filtered with High Efficiency Particulate Air (HEPA) class H14 air filters in all five OTs under evaluation.

**Figure 1:**
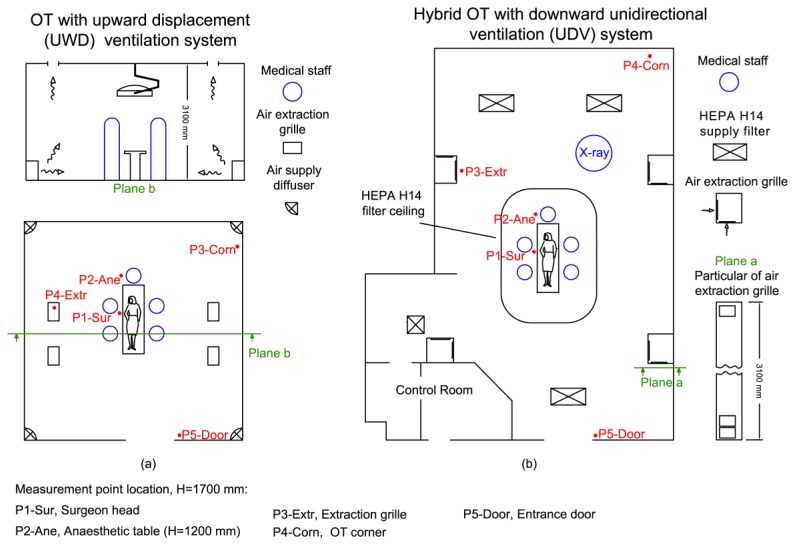
Operating theatres and sampling probe layout position: (**a**) OT with upward displacement airflow (UWD) ventilation system; (**b**) Hybrid OT with unidirectional downward airflow (UDV) ventilation system.

Each OT adopting UWD ventilation principle is equipped with four air supply diffusers located at floor level, and four extraction grilles placed at the ceiling level (Figure 1a). The recovery time (average value of the four UWD OTs), in at-rest condition, measured outside the critical area and close to the extraction grilles is equal to 280 s. The hybrid OT adopting UDV ventilation system (see Figure 1b), is equipped with a HEPA H14 filtration ceiling of 12 m^2^, positioned above the critical surgical area located at the room center, with seven additional HEPA H14 filters supplying air in the remaining room area external to the critical zone. Air extraction grilles are positioned along the peripheral walls according to the scheme in Figure 1b. The measured recovery time, in at-rest condition and outside of the unidirectional airflow zone (point P3-extr in Figure 1b) is equal to 120 s.

The design value of airflows and of pressure difference between the OTs and the adjacent premises, equal to 10 Pa, were maintained in all OTs during the entire experimental campaign. In all the five OTs, a fraction equal to 70% of the supply airflow rate (Table 1) is recirculated air while the remaining 30% is fresh air. Indoor air temperature and humidity were kept at the set point (20 °C and 55% RH). UFPs and FPs, in the size range from 0.02 up to 1 μm, have been measured with specific instruments (see below) in selected positions; the reported temperature, humidity and pressure data are mean values obtained by the OT monitoring and control system.

### 2.2. Instruments Description and Location

UFP concentrations have been monitored by two ultrafine particle counters (UFP-C, P-Trak mod. 8525, TSI Inc., Shoreview, MN, USA). UFP-C is based on a condensation particle counter (CPC) technique which can detect and count cumulative particle concentration in the range of 0.02 up to 1 μm. The concentration limit is 5 × 10^5^ pp/cm^3^ and the sampling flow rate is 0.1 L/min, according to ISO 27891 [60], with an accuracy of 15% of the reading measurement. Calibration data of measured concentration in particles/cm^3^, based on instruments certificate, show a deviation of ±4%. The relative error between the two UFP-Cs used was less than 5%. Measurements have been carried out simultaneously at two different locations per every surgical operation.

The airborne particle sampling locations have been chosen in consideration of the most relevant medical staff positions during surgeries through a preliminary on-site analysis. Therefore, the selected sampling locations were positioned (see Figure 1) as follows: (a) close to the surgeon head (P1-Sur); (b) close to the anesthetic table (P2-Ane); (c) close to an air extraction grille (P3-Extr); (d) close to one room corner (P4-Corn); (e) close to the entrance door (P5-Door). The air sampling probes of the UFP-Cs have been placed at a height of 1.7 m, with exception of point P2-ane, which was placed at 1.2 m throughout the entire measurement campaign.

### 2.3. Experimental Procedure

In order to have a continuous measurement during the surgical operations and to detect the UFP peak levels, the sampling time of UFP counters was set equal to 5 s, with no hold-on time. Ten different surgical operations, denoted with letters A–J, have been monitored. Surgical teams, in all the monitored surgeries, were dressed in surgical clothes with low particle and Colony-Forming Units (CFUs) release, according to previous work of Romano et al. [61]. Monopolar, bipolar or argon diathermy were the three ESTs techniques used during the monitored operations and, for sake of comparison between different surgical operations, the use of ESTs and their usage time were as similar as possible.

Among the ten (10) real operations (A–J) under evaluation, liver resection (operations denoted with letters A–F) was the most recurrent type of surgery monitored. Liver resection surgery is a routine procedure acting on a target tissue which produces large quantities of surgical smoke. Liver surgeries have been carried out both in OTs equipped with UWD and with UDV ventilation principles; they can be considered similar and comparable procedures, irrespective of patient differences. Different surgeries, still comparable in terms of surgical procedures, i.e., Whipple pancreas removal procedure, or pancreaticoduodenectomy (operation G), removal of gallstone (operation H), and skin cancer (operations I–J) have also been monitored.

As reported by Steege et al. [28], when ESTs are in use a large part of the medical staff, and in particular surgeons, are exposed to surgical smoke within five feet of the source, i.e., the area close to the surgical table and patient. This distance—five feet—can be used in order to determine the region occupied by personnel during surgeries and to define the breathable zone as suggested by ASHRAE [43]. Let us point out that there are no specific limits values set by OSHA standards for laser and electrosurgery contaminant plume hazards. OSHA [1] and NIOSH [45] recommend to adopt preventive measures and protective equipment against surgical smoke exposure. Therefore, the measurements of UFP contaminant concentration and of exposure time, in the positions where medical staff is exposed (i.e., at the surgeon, P1-Sur, and at the anesthesiologist position, P2-Ane) and during real ongoing activities, seem to be representative and comparative parameters to evaluate the amount of surgical smoke to which personnel are exposed. The experimental data obtained in the positions where surgical staff is exposed, are used in order to make comparisons between the two ventilation systems under evaluation and they could give useful indications on their relative capability to remove surgical smoke and to reduce worker exposure.

The other sampling locations (P3–P5) have been measured only when the occurrences have not allowed measurement in the two main sampling points close to the critical area (P1-Sur and P2-Ane). These results are useful in extending the knowledge on the airflow conditions and on the contaminant dispersal. In the experimental campaign encompassing different real surgeries, the number and the qualification of the medical staff, and the type of ESTs used have been considered and recorded.

### 2.4. Ethics

The experimental campaign took place in a Swedish hospital. Swedish legislation (Act 2003:460, Amended SFS 2008:192) does not demand ethical permission for these types of observational studies that do not involve patients. However, informed consent in line with the Declaration of Helsinki was given to all OT teams (World Medical Association, 2013). Moreover, the medical person in charge from the university hospital was involved in the research work.

## 3. Results and Discussions

This work highlights how various medical activities can be carried out differently within an OT environment in which the number and the composition of medical staff is continuously changing, e.g., surgeons, surgical nurses, nurses, anesthesiologists, and radiologists. Their positions within the OT depend on the type of surgery and occurrences, e.g., surgeon close to the surgical table, anesthesiologists on the tip of the table and nurses beside surgeons and so on. Moreover, medical equipment, depending on the surgery, can be placed in different positions.

This experimental measurement campaign has been carried out with the aim of collecting and analyzing data about surgical smoke contamination during real surgeries in common and hybrid OTs equipped with two different ventilation schemes and taking into account conditions and parameters that could affect the contaminant generation and removal and the personnel exposure.

The largest presence of personnel during ongoing activities was reached during liver resection surgery in the UDV OT with 14 surgical staff present in the OT at the same time, while the recorded minimum value was four persons and occurred in the OT equipped with UWD system. Table 2 shows the average values of medical staff composition during monitored liver resection surgeries for the two OT types and ventilation principles (hybrid OT with UDV and common OT with UWD).

The larger plan area and the higher air supply volume together with the shorter recovery time of the hybrid OTs with a UDV system (see Table 1), allow the presence of a larger number of staff members during surgeries as compared with OTs with a UWD system (Table 2).

In the ten surgical activities monitored (A–J), three types of electrosurgical instruments (monopolar, bipolar and argon diathermy) were used at least once for a minimum period of 1 min, a maximum of 15 and an average value of 4. ESTs were used on average for 30% of the entire operation time during all surgeries monitored.

The ventilation principles used in the OTs during the 10 surgeries (A–J) are shown in Table 3 as well as the time average UFP concentration measured.

Sampling points P1-Sur or P2-Ane, are representative of the highest contamination exposure level around the surgical table where there is also the highest medical staff density.

The analysis of the experimental measurement carried out has shown that the activity of monopolar, bipolar and argon diathermy electrosurgical tools has a strong influence on the airborne contamination level in the OT. The type of ESTs adopted and their working time create a noteworthy increase of UFP concentration close the target tissue which also influences the entire OT area. The surgical smoke exposure, in terms of UFP concentration, experienced by the medical staff within the critical area and the entire OT environment is a direct consequence of the type of ventilation principle system adopted, the surgical activity, the number of personnel and obstacles present in the OT.

**Figure 2:**
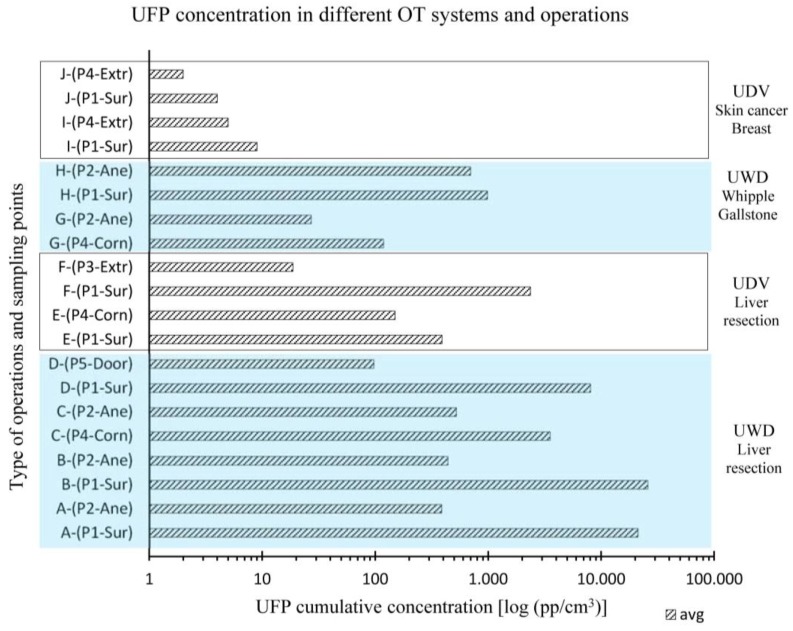
UFP cumulative time average concentration values vs. OT and ventilation system type (UDV or UWD), surgery activity and sampling point positions (P1-Sur to P5-Door) for different surgical activities (operations A to J).

Figure 2 shows the time average concentration values of UFPs measured during the ten surgeries monitored (A–J) in different sampling locations (P1-Sur to P5-Door). Results are also grouped by the type of surgical operation (liver, skin cancer, etc.) conducted and the specific ventilation principle (UWD and UDV) used in the OTs.

Based on the experimental measurements conducted, surgeons who have carried out a standard liver resection surgery in the OT equipped with UWD system have experienced, on average throughout the entire operation, an exposure to surgical smoke 13 times higher than in the OT equipped with the UDV system.

The use of ESTs strongly influences the airborne contamination within the OT environment. Activities with limited usage of ESTs, as for example gallstone, whipple and skin cancer operations (G to J), implicate a lower UFP concentration. In this case, operations I and J, carried out in OTs equipped with UDV ventilation, had lower UFP concentration values as compared with the operations G and H carried out in OTs equipped with UWD systems.

Results show large variations in the airborne contaminants distribution within the OT environment during surgical operation. A high level of surgical smoke has been detected both in the critical area close to the surgical table as well as in the different locations within the theatre. Moreover, the two ventilation schemes in the evaluated OTs lead to different contamination levels of UFPs within the environments (point P1-Sur to P5-Door) in all operations monitored (A to J).

However, the measured UFP concentrations, points P1-Sur to P5-Door, highlight that all the staff categories are exposed to surgical smoke in both types of OT and ventilation schemes adopted, but with large differences in concentration. The UDV principle based OTs can achieve lower UFP concentration both inside and outside of the critical area. In fact, in OTs with downward unidirectional airflow, a stable and well-defined airflow pattern sweeps away from the critical area the internal contamination generated, e.g., surgical smoke. Moreover, the high air velocity of the airflow pattern along the peripheral area of the ceiling filter and the location of the extraction grilles impede the entrainment of the external contamination. This combined effect provides a rapid decontamination inside and outside the critical area as also confirmed by the lower recovery time of this system in comparison with the UDW system, as also shown by Romano et al. [62].

On the contrary, the ventilation principle based on the upward displacement system has the characteristic of using lower air volume with the displacement air distribution pattern, i.e., from the floor to the ceiling. However, the low air velocity, the reduced airflow rate and the contamination entrained from the floor, make this ventilation scheme less effective in protecting people staying in the critical area around the surgical table, and in particular during the use of ESTs.

## 4. Conclusions

Surgical smoke exposure has been linked to adverse acute health effects in exposed healthcare workers. There is a lack of standards and precise guidelines for this issue. The monitoring of the surgical smoke generated by ESTs in terms of UFP concentration in the critical area around surgical tables may give a reasonable value of the exposure level to which medical staff is exposed during real surgeries. In this research work, the surgeon and anesthetic site, respectively P1-Sur and P2-Ane, have been chosen as the critical points for measuring the exposure level. The effect of the ventilation system installed in operating theatres, the airflow rate, the type and the usage time of ESTs as well as the personnel presence in the OTs have been evaluated in this work.

Different types of surgical operations have been studied and documented. The results obtained have been used to compare the exposure of medical staff to surgical smoke as a function of the ventilation type adopted in the OTs under evaluation; more specifically, in a hybrid OT with UDV ventilation and common OTs with UWD. The experimental results have confirmed that UFP concentrations, and therefore the personnel exposure, are strictly dependent on the usage of ESTs and on the type of surgical activity carried out. However, the ventilation system adopted plays an important role. The comparative studies conducted on the ten surgeries monitored indicate that the hybrid OT equipped with the UDV ventilation system had UFP concentration values of surgical smoke lower than the OTs adopting the UWD system. In the case of liver resection surgery, at sampling point P1-Sur, UFP concentrations in the UDV OT were on average 13 times lower than in surgeries conducted in UWD OTs. Similar trends have been observed for a whipple pancreas removal procedure and for skin cancer surgeries. The difference in UFP concentrations, in the different OT and in the monitored surgeries, can be ascribed to the different ventilation systems adopted.

In the specific cases discussed in this work, the UDV system with its large airflow volume and well-defined airflow pattern evacuates the surgical smoke nearby the critical area faster and more efficiently than OTs with UWD ventilation systems, as also demonstrated by its short recovery time. The position of the extraction grilles in the UDV system are well-defined and localized in order to achieve the best evacuation effect without interfering with obstacles within the critical area. On the contrary, in the UWD system layout presented in this work, the extraction grilles facilitate the entrainment and recirculation of contaminants just above the breathing zone of the critical area.

In order to provide surgical staff with adequate ventilation for contaminant removal, and therefore for decreased levels of personnel exposure to surgical smoke, OT environments should be provided with large airflow volume at reasonable air velocity and with well-defined airflow paths both at the in inlet and at the outlet sections. Moreover, ventilation design should carefully consider the real operating conditions existing during practical surgeries. Medical staff involved in surgical processes should be more aware of how their behavior can affect the surgical smoke dispersion and control, and must use LEV and PPE equipment. Future studies must deal with the microbiological contamination of surgical smoke over OT surfaces, personnel garments and the related infection risks.

## Figures and Tables

**Table 1:** Technical data of evaluated OTs: four with UWD and one hybrid with UDV ventilation.

Ventilation Principle	Area (m²)	Volume (m³)	Supply Air (m^3^/h)	Extraction Air (m^3^/h)	Air Changes per Hour (1/h)	Surgical Lamps
UWD	37	100	1.998	1.328	20	2
UDV	100	270	15.480	15.120	57	2

OTs, operating theatres; UWD, upward displacement airflow; UDV, unidirectional downward airflow.

**Table 2:** Composition of the medical staff during six liver resection operations (A to F) in different OT types and ventilation principles.

OT Type & Ventilation	Operation Type	Surgeons	Nurses	Anesthetist	Visitors	Xray Nurse	Total Staff
UDV	Liver resection	3	4	1	0	1	9
UWD	Liver resection	2	3	1	0	0	6

**Table 3:** Type of OT ventilation systems and surgeries during the measuring campaign. Time average number of UFP concentrations (pp/cm^3^) detected close to the surgical table at the surgeon site (P1-Sur) during different surgical operations (standard deviation within brackets).

Items	Surgical Operation					
	Liver Resection	Liver Resection	Skin Cancer	Breast Gland	Gallstone	Whipple *
Type of OT & Ventilation system	UWD	UDV	UDV	UDV	UWD	UWD
N° surgeries monitored	4	2	1	1	1	1
Total hours monitored (h)	14	12	0.7	0.7	2.7	3.8
UFP 0.02–1 μm (pp/cm^3^)	18.284 (65.500)	1.380 (1.800)	9 (20)	4 (3)	981 (2200)	27 (60)

* UFP (ultrafine particles) concentration measured at point P2-Ane.
